# Introduction: the 13th annual conference of The Australia & New Zealand Academy for Eating Disorders

**DOI:** 10.1186/2050-2974-3-S1-I1

**Published:** 2015-11-23

**Authors:** Kim Hurst, Jeremy Freeman

**Affiliations:** 1 Child, Youth and Adult Eating Disorder Programme, Gold Coast, Australia; 2 Australia & New Zealand Academy of Eating Disorders, Sydney, Australia

## 

The 13^th^ annual conference of the Australia & New Zealand Academy for Eating Disorders (ANZAED) "*Riding the Waves to Recovery*" was held on 21-22 August 2015 at the Surfers Paradise Marriott Resort & Spa Hotel, Gold Coast, Queensland, Australia. Over 300 delegates attended the conference from all over the world, presenting 72 Oral papers and 26 Posters following peer review by the Scientific Committee. Most of these are published in this supplement.

The international keynote speaker was Professor Ivan Eisler from the Institute of Psychiatry, Kings College London. Professor Eisler discussed *“Treatment models, brand names and evidence-based practice in eating disorders: should we be treating people by the book?*”. Professor Stephen Touyz from the University of Sydney was the second keynote speaker: *“Grappling with the critical factors of severe and enduring anorexia nervosa (SE-AN): Complex issues requiring novel ideas”.*

The symposium: *“The Crescendo of Hope”* combined the perceptions and experiences of Sarah Louise McKenzie (person in recovery), David Homewood (father of an adolescent in recovery) and Lisa Dawson (researcher and clinician). The other Plenary: *“Weighing & Weight as part of the Treatment and Recovery from Anorexia Nervosa*” was explored by Dr Alexia Murphy (Research Fellow at the Children’s Nutrition Research Centre), Dr Michael Kohn (Clinical Associate Professor in the Faculty of Medicine at Sydney University, and paediatrician Sydney Children’s Hospital Network) and Dr. Linsey Atkins (Clinical Psychologist and Director of Hope Family Clinic).

“*Hot Off The Press*” highlighted Associate Professor Sue Byrne’s principle results from the “Strong without Anorexia Nervosa (SWAN) Study: A Multicentre Randomised Controlled Trial of Three Psychological Treatments for Anorexia Nervosa in Adults” and Professor Phillipa Hay, Editor-in-Chief – Journal of Eating Disorders discussed the unique benefits of Open Access publication

The 2015 ANZAED Lifetime Achievement Award was presented to Professor Phillipa Hay, and the 2015 ANZAED Distinguished Achievement award was presented to Eating Disorder Association of South Australia (EDASA) co-founders Loraine House and Kate Parsons.

